# GP73 represses host innate immune response to promote virus replication by facilitating MAVS and TRAF6 degradation

**DOI:** 10.1371/journal.ppat.1006321

**Published:** 2017-04-10

**Authors:** Xuewu Zhang, Chengliang Zhu, Tianci Wang, Hui Jiang, Yahui Ren, Qi Zhang, Kailang Wu, Fang Liu, Yingle Liu, Jianguo Wu

**Affiliations:** State Key Laboratory of Virology and College of Life Sciences, Wuhan University, Wuhan, P. R. China; University of North Carolina at Chapel Hill, UNITED STATES

## Abstract

Hepatitis C virus (HCV) infection is a leading cause of chronic liver diseases and hepatocellular carcinoma (HCC) and Golgi protein 73 (GP73) is a serum biomarker for liver diseases and HCC. However, the mechanism underlying GP73 regulates HCV infection is largely unknown. Here, we revealed that GP73 acts as a novel negative regulator of host innate immunity to facilitate HCV infection. GP73 expression is activated and correlated with interferon-beta (IFN-β) production during HCV infection in patients’ serum, primary human hepatocytes (PHHs) and human hepatoma cells through mitochondrial antiviral signaling protein (MAVS), TNF receptor-associated factor 6 (TRAF6) and mitogen-activated protein kinase kinase/extracellular regulated protein kinase (MEK/ERK) pathway. Detailed studies revealed that HCV infection activates MAVS that in turn recruits TRAF6 *via* TRAF-interacting-motifs (TIMs), and TRAF6 subsequently directly recruits GP73 to MAVS *via* coiled-coil domain. After binding with MAVS and TRAF6, GP73 promotes MAVS and TRAF6 degradation through proteasome-dependent pathway. Moreover, GP73 attenuates *IFN-β* promoter, IFN-stimulated response element (ISRE) and nuclear factor κB (*NF-κB*) promoter and down-regulates *IFN-β*, *IFN-λ1*, interleukin-6 (*IL-6*) and IFN-stimulated gene 56 (*ISG56*), leading to the repression of host innate immunity. Finally, knock-down of *GP73* down-regulates HCV infection and replication in Huh7-MAVSR cells and primary human hepatocytes (PHHs), but such repression is rescued by GP73m4 (a mutant GP73 resists to GP73-shRNA#4) in Huh7-MAVSR cells, suggesting that GP73 facilitates HCV infection. Taken together, we demonstrated that GP73 acts as a negative regulator of innate immunity to facilitate HCV infection by interacting with MAVS/TRAF6 and promoting MAVS/TRAF6 degradation. This study provides new insights into the mechanism of HCV infection and pathogenesis, and suggests that GP73 is a new potential antiviral target in the prevention and treatment of HCV associated diseases.

## Author summary

Golgi protein 73 (GP73) is a serum biomarker for liver diseases and hepatocellular carcinoma (HCC). In this study, the authors reveal that GP73 acts as a novel negative regulator of host innate immunity to facilitate hepatitis C virus (HCV) infection. GP73 expression is activated and correlated with IFN-β production during HCV infection in patients’ serum, primary human hepatocytes (PHHs) and human hepatoma cells through mitochondrial antiviral signaling protein (MAVS), TNF receptor-associated factor 6 (TRAF6) and MEK/ERK pathway. They further demonstrate that during viral infection, MAVS recruits TRAF6 that subsequently directly binds with GP73. After binding with MAVS and TRAF6, GP73 promotes MAVS and TRAF6 degradation. Moreover, GP73 attenuates *IFN-β* promoter, IFN-stimulated response element (ISRE) and *NF-κB* promoter and down-regulates *IFN-β*, *IFN-λ1*, interleukin-6 (*IL-6*) and IFN-stimulated gene 56 (*ISG56*), leading to the repression of host innate immunity and the facilitation of virus infection. These results reveal a novel mechanism by which GP73 acts as a novel negative regulator of host innate immunity to facilitate virus infection and also provide new insights into the therapeutic design of anti-HCV drugs.

## Introduction

Innate immune response is critical for host defense against microbial infection including bacteria, fungi and viruses. Upon microbial infection, pathogen-associated molecular patterns (PAMPs) are recognized by pattern recognition receptors (PRRs), which lead to the production of type I interferons (IFNs), proinflammatory cytokines and downstream effectors [1]. Among PRRs, Toll-like-receptors (TLRs) and RIG-I-like receptors (RLRs) recognize viral RNAs. Certain TLRs detect viral RNA in endosome, such as TLR3 senses viral double-stranded RNA (dsRNA) and TLR7/8 recognizes single-stranded RNA (ssRNA) [2, 3]. While RLRs, including retinoic acid inducible gene I (RIG-I) and melanoma differentiation-associated gene 5 (MDA5), sense viral RNA in the cytoplasm, which in turn recruit and activate mitochondrial antiviral signaling protein (MAVS) [4–7]. MAVS further recruits TNF receptor associated factors (TRAF2/3/5/6) to activate nuclear factor κB (NF-κB) and interferon regulatory factors (IRF3/7) leading to the production of IFNs and cytokines [1, 8, 9].

Hepatitis C virus (HCV) infection is a major cause of chronic liver diseases, chronic hepatitis, fibrosis and cirrhosis, which have a marked risk of developing hepatocellular carcinoma (HCC) [10]. HCV contains a 9.6-kb positive-sense RNA genome encoding a 3000-amino acids polyprotein that is cleaved into four structural proteins (Core, E1, E2 and P7) and six nonstructural proteins (NS2, NS3, NS4A, NS4B, NS5A and NS5B) [11]. NS3/4A protease is essential for generating mature proteins required for virus replication and abrogating host antiviral innate immunity by cleaving MAVS and TIR-domain-containing adaptor-inducing IFN-β (TRIF) [5, 12, 13]. Previous studies showed that *in vitro* transcribed HCV genomic RNA and 3’untranslated region (3’UTR) of RNA are recognized by RIG-I to trigger IFN response [14, 15]. Recent study reported that MDA5 plays a major role in IFN response during HCV infection by introducing a mutant MAVS (MAVS-C508R, resistant to NS3/4A cleavage) into human hepatoma Huh7 cells [16].

Golgi protein 73 (GP73) is a resident Golgi membrane protein initially identified in adult giant-cell hepatitis [17]. It is constitutive expressed in normal livers, but up-regulated in liver diseases [17, 18]. Clinical reports showed that GP73 is a novel HCC serum marker with high specificity and sensitivity [19–23]. HCV facilitates GP73 expression that in turn enhances HCV secretion [24]. Mammalian target of rapamycin complex-1 (mTORC1) up-regulates GP73 that subsequently promotes HCC cell proliferation and xenograft tumor growth in mice [25]. However, the mechanism by which GP73 regulates HCV infection and pathogenesis is largely unknown.

Here, we revealed a novel mechanism by which GP73 facilitates HCV infection through repressing IFN signaling. Initially, HCV infection activates GP73 in patients’ serum, primary human hepatocytes (PHHs) and human hepatoma cells by regulating MAVS/TRAF6 and MEK/ERK pathway. Subsequently, GP73 binds with MAVS/TRAF6 to promote MAVS and TRAF6 degradation by proteasome-dependent pathway, which leads to the repression of host innate immunity and facilitation of HCV infection.

## Results

### GP73 expression is activated and correlated with IFN activation during HCV infection

**Fig 1:**
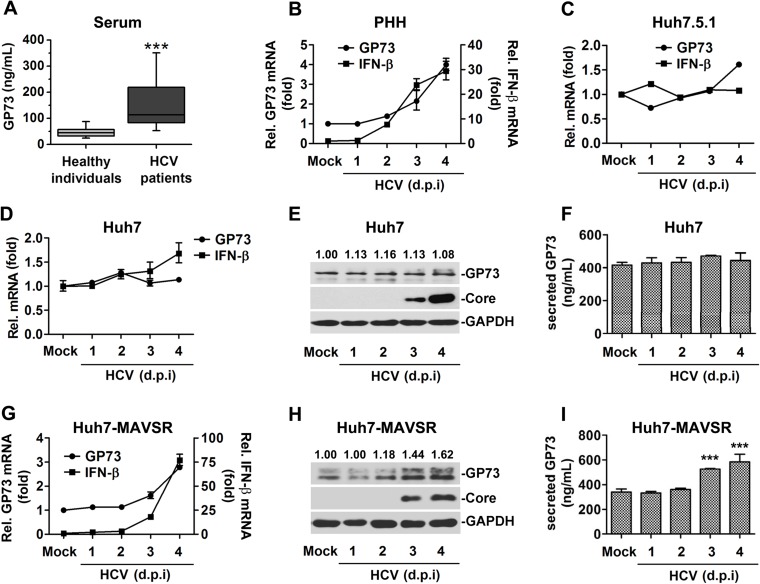
GP73 expression is activated and correlated with IFN activation during HCV infection. (**A**) Serum GP73 levels in HCV-infected patients (n = 60) and healthy individuals (n = 60) were detected by ELISA. Samples were tested in duplicate and concentrations were determined from standard curves. Data represent means ± SEM. Boxplots illustrate medians with 25% and 75% and error bars for 5% and 95% percentiles. (**B, C**) Primary human hepatocytes (PHHs) (**B**) or Huh7.5.1 cells (**C**) were infected with HCV at a multiplicity of infection (MOI) of 2 for indicated times. *GP73* and *IFN-β* mRNAs were quantified by RT-PCR. (**D**, **E**, and **F**) Huh7 cells were infected with HCV at MOI = 2 for indicated times. The mRNA levels of *GP73* and *IFN-β* were determined by RT-PCR (**D**), the protein levels of GP73 were determined by WB (**E**) and ELISA (**F**). (**G**, **H**, and **I**) Huh7-MAVSR cells were infected with HCV at MOI = 2 for indicated times. The mRNA levels of *GP73* and *IFN-β* were determined by RT-PCR (**G**), the protein levels of GP73 were determined by WB (**H**) and ELISA (**I**). Bar graphs represent means ± SD, ***P < 0.001, compared with control group. (d.p.i, days post infection).

The effect of HCV infection on GP73 expression was initially investigated. First, secreted GP73 protein was determined in the serum of HCV-infected patients (n = 60) and healthy individuals (n = 60) (Table 1). Serum GP73 protein was significantly higher in HCV infected patients compared to healthy individuals (mean ± standard error of the mean [SEM] 161±14.2 versus 47.6 ±2.6 ng/ml) (Fig 1A), suggesting that GP73 is activated in infected patients. Second, *GP73* mRNA was determined in primary human hepatocytes (PHHs) infected with HCV (JFH1 HCVcc). *GP73* and *IFN-β* mRNAs were up-regulated by HCV (Fig 1B), indicating that *GP73* is activated and correlated with *IFN-β* during HCV infection. Third, *GP73* mRNA was determined in Huh7.5.1 and Huh7 cells infected with JFH1 HCVcc. To our surprise, *GP73* and *IFN-β* mRNAs were relatively unchanged (less then 1.5-fold) in HCV-infected cells (Fig 1C and 1D), the protein levels of GP73 were also unchanged in HCV-infected Huh7 cells (Fig 1E and 1F), suggesting that GP73 is not activated by HCV in Huh7.5.1 and Huh7 cells with the defective in IFN response in the cells.

**Table 1:** Baseline characteristics of HCV-infected patients and healthy individuals.

Characteristic	Healthy individuals (N = 60)	Patients (N = 60)
Age (years)	48.4±16.1	52.4±13.6
Gender (male/female)	28/32	34/26
AST (U/L)	<40	58.8±5.4
ALT (U/L)	<30	66.0±7.0
HCV RNA (copies/ml)	<500	1.4E+07±3.3E+06
GP73 (ng/ml)	47.6±2.6	161.0±14.2***

Data are presented as mean ± SEM

***p < 0.001 compared with Healthy individuals group; Abbreviations: AST, aspartate aminotransferase; ALT, alanine aminotransferase.

It was reported that MDA5 senses HCV RNA to trigger IFN response after introducing a C508R mutant of MAVS (MAVSR) into Huh7 cells [16]. We next investigated the expression status of *GP73* and *IFN-β* in HCV-infected Huh7-MAVSR cells. The results demonstrated that in Huh7-MAVSR cells, *GP73* and *IFN-β* mRNAs were up-regulated by at least 3-fold during HCV infection (Fig 1G), the protein levels of GP73 were also significantly enhanced during HCV infection in Huh7-MAVSR cells (Fig 1H and 1I). We also showed that GP73 and IFN-β were activated by other RNA viruses, vesicular stomatitis virus (VSV) and enterovirus 71 (EV71) (S1A and S1B Fig). These results demonstrated that GP73 production is activated and correlated with IFN-β activation during viral infection, probably through MAVS.

After MAVS activation during viral infection, IRF3/NF-κB is stimulated to induce IFN, whereas TAK1/MAPK is also activated to modulate cell proliferation. Thus, we determined which pathway is involved in GP73 activation. *GP73* mRNA level was not affected by the treatments of recombinant IFN-α or recombinant IFN-λ1, suggesting that *GP73* is not an interferon-stimulated gene (ISG) (S1C Fig). *GP73* mRNA was repressed by PD98059 and U0126 (MEK/ERK inhibitors), but not by GF109203, LY294002, SP600125, SB203580 or BAY11-7082 (PKC, PI3K, JNK, P38 or NF-κB inhibitors) (S1D Fig), and *GP73* mRNA was attenuated by PD98059 and U0126 in dose-dependent manners (S1E Fig), suggesting that *GP73* expression is modulated by MEK/ERK pathway, but not PKC, PI3K, JNK, P38 or NF-κB pathways. Since mTORC1 pathway activates GP73 in HCC [25], we examined the effect of rapamycin (mTORC1 inhibitor) on *GP73* expression. *GP73* mRNA was not affected by rapamycin (S1F Fig), but GP73 protein was repressed by rapamycin or U0126 (S1G Fig), suggesting that *GP73* transcription is dependent on ERK pathway. Thus, GP73 expression is activated and correlated with IFN activation through MAVS and probably through MAVS-mediated MEK/ERK pathway during HCV infection in human serum, primary human hepatocytes and human hepatoma cells.

### GP73 represses host innate immunity during viral infection

**Fig 2:**
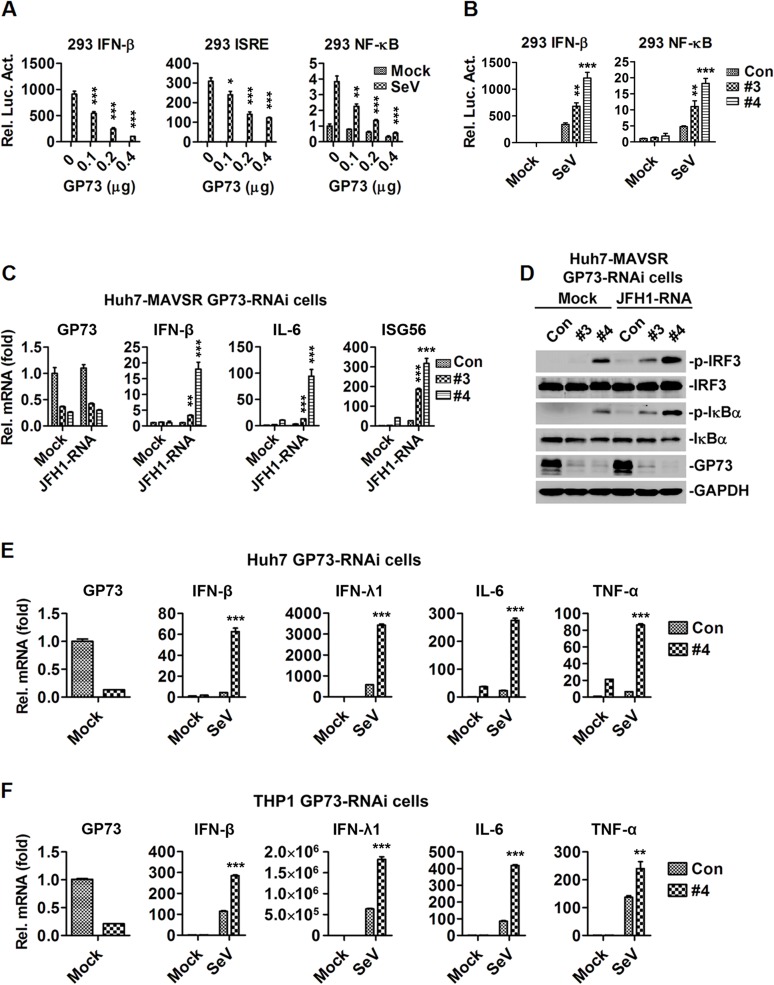
GP73 represses host innate immunity during viral infection. (**A**) HEK293 cells (1×10^5^) were transfected with the *IFN-β*, *ISRE* or *NF-κB* reporters (0.1 μg) and *GP73* expressing plasmid at indicated concentrations for 24 h, and infected with SeV for 10 h before luciferase reporter assays were performed. (**B**) HEK293 cells (1×10^5^) were transfected with *IFN-β* or *NF-κB* reporter (0.05 μg) and *GP73*-shRNAs expressing plasmids (1 μg) for 48 h, and infected with SeV for 10 h before reporter assays were performed. (**C**, **D**) *GP73*-shRNAs transduced stable Huh7-MAVSR cells were transfected with *in vitro* transcribed JFH1 genomic RNA for 16 h. *IFN-β*, *IL-6* and *ISG56* mRNAs were quantified by RT-PCR (C) and p-IRF3 and p-IκBα were detected by WB (D). (**E**, **F**) *GP73*-shRNAs transduced stable Huh7 (**E**) and THP-1 (**F**) cells were infected with SeV for 12 h. *IFN-β*, *IFN-λl*, *IL-6* and *TNF-α* mNRAs were quantified by RT-PCR. Bar graphs represent means ± SD, *P < 0.05, **P < 0.01, ***P < 0.001, compared with control group.

Because GP73 production is correlated with IFN activation, we evaluated the effect of GP73 on the regulation of IFN signaling in HEK293 cells transfected with IFN-β-Luc, ISRE-Luc or NF-κB-Luc reporters and infected with SeV. IFN-β-Luc, ISRE-Luc and NF-κB-Luc were activated by SeV, but such activation was attenuated by GP73 (Fig 2A), indicating that GP73 represses IFN-β, ISRE and NF-κB activities. GP73 contains an N-terminal trans-membrane (TM) domain required for membrane localization, a C-terminal acidic tail, and a middle coiled-coil domain required for endosome-to-Golgi traffic [26–28]. Based on the functional domains, we constructed a series of truncations of GP73 (S2A Fig) and examined the effects of mutants on the regulation of *IFN-β*. Activation of *IFN-β* reporter mediated by SeV was repressed by GP73, D1 (1–348) and D2 (1–205), enhanced by D4 (56–401), D5 (56–348) and D6 (56–205), but not affected by D3 (1–55) or D7 (206–401) (S2B Fig), indicating that TM domain plus coiled-coil domain, but not coiled-coil domain alone, repress SeV-mediated activation of *IFN-β*. These results also implicated that inhibitory effect of GP73 dependents on Golgi membrane targeting or sub-cellular compartments trafficking. Further expression analysis showed the protein levels of GP73 D1 and GP73 D2 were comparable to FL GP73, but GP73 D4 and GP73 D6 were lower than FL GP73 (S2C Fig), suggesting the TM domain plus coiled-coil domain were sufficient to inhibit *IFN-β* activation, and that the expression level of *GP73* may affect its repression effect on *IFN-β* activation.

The role of endogenous GP73 in virus-triggered IFN signaling was examined by three shRNAs targeting to *GP73* gene (*GP73*-shRNA#2, #3 and #4), which could attenuate *GP73* mRNA and GP73 protein level (S2D Fig). *IFN-β* and *NF-κB* activities were induced by SeV and further facilitated by *GP73*-shRNA #3 and *GP73*-shRNA#4 in HEK293 cells (Fig 2B), indicating that knock-down of *GP73* up-regulates virus-induced *IFN-β* and *NF-κB*.

The effect of GP73 on the regulation of innate immunity was assessed. We initially showed that *GP73* mRNA was highly expressed in Huh7 and Huh7.5.1 cells, at low levels in L02, HEK293, THP-1 and HeLa cells, but not detected in HepG2 cells (S2E Fig). Thus, we generated three stable cell lines: Huh7-MAVSR/lentivirus-Con-shRNA, Huh7-MAVSR/lentivirus-GP73-shRNA#3 and Huh7-MAVSR/lentivirus-GP73-shRNA#4. IFN-β, IL-6 and IFN-stimulated gene 56 (ISG56) were enhanced by GP73-shRNA#3 and GP73-shRNA#4 in the presence of JFH1 HCV genomic RNA (Fig 2C), indicating that knock-down of GP73 up-regulates IFN-β, IL-6 and ISG56. In addition, p-IRF3 and p-IκBα were facilitated by GP73-shRNA#3 and GP73-shRNA#4 in the presence of HCV genomic RNA (Fig 2D), suggesting that knock-down of *GP73* enhances IRF3 and IκBα phosphorylation. Moreover, *IFN-β*, *IFN-λ1*, *IL-6* and *TNF-α* mRNAs were activated by SeV in the presence of *GP73*-shRNA #4 (Fig 2E and 2F), indicating that knock-down of *GP73* facilitates *IFN-β*, *IFN-λ1*, *IL-6* and *TNF-α* expression. Furthermore, *IFN-β*, *IFN-λ1*, *IL-6* and *ISG56* mRNAs were also activated by SeV in the presence of *GP73*-shRNA #4, compared with a seed sequence-matched control (S2F Fig), indicating that the effect was not due to non-specific off-targets of *GP73*-shRNA#4. Thus, GP73 acts as a negative regulator to repress host innate immunity during viral infection.

### GP73 interacts and co-localizes with MAVS and TRAF6

**Fig 3:**
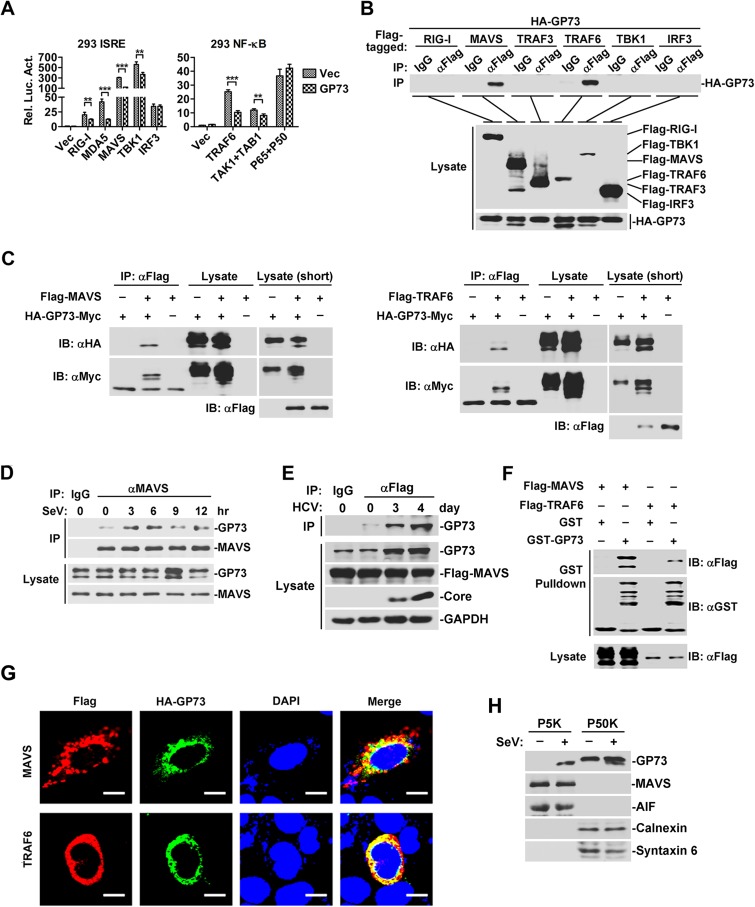
GP73 interacts and co-localizes with MAVS and TRAF6. (**A**) HEK293 cells (1×10^5^) were co-transfected with *GP73* expressing plasmid (0.1 μg) and *ISRE* or *NF-κB* reporters (0.05 μg) along with other plasmids as indicated (0.1 μg). Luciferase assays were performed 20 h after transfection. (**B**) HEK293 cells (2×10^6^) were co-transfected with *GP73* expressing plasmid (1 μg) and plasmids as indicated (3 μg) for 24 h. Cells were lysed and lysates were immunoprecipitated with anti-Flag or IgG. Immunoprecipitates and whole cell lysates (WCLs) were analyzed by WB with anti-HA or anti-Flag. (**C**) HEK293 cells (2×10^6^) were co-transfected with HA-*GP73-Myc* (2 μg) and Flag-*MAVS* or Flag-*TRAF6* (3 μg) for 24 h. Cells were lysed and lysates were immunoprecipitated with anti-Flag. Immunoprecipitates and WCLs were analyzed by WB with indicated antibodies. (**D**) HEK293 cells (2×10^7^) were infected with SeV for indicated times. Immunoprecipitation and WB analysis were performed with antibodies against indicated proteins. (**E**) Huh7-MAVSR cells (5×10^7^) were infected with HCV (MOI = 2) for the indicated days. Immunoprecipitation and WB analysis were performed with antibodies against indicated proteins. (**F**) HEK293 cells (5×10^5^) were transfected with plasmids as indicated (2 μg) for 24 h. Cell lysates were subjected to GST pull-down assays with equal molar quantity of purified GST (10 μg) or recombinant GST-GP73 (20 μg) proteins. Immunoblots were performed with indicated antibodies. (**G**) HeLa cells (1×10^5^) were co-transfected with HA-tagged *GP73* plasmid (0.3 μg) and Flag-tagged *MAVS* or *TRAF6* plasmids (0.7 μg) for 36 h. Cells were fixed and stained with a rabbit anti-HA monoclonal antibody (MAb) and a mouse anti-Flag MAb, followed by the FITC-conjugated goat anti-rabbit secondary antibody and a DyLight649-conjugated goat anti-mouse secondary antibody before confocal microscopy analysis. Nuclei were counterstained with DAPI. Scale bar, 10 μm. (**H**) HEK293 cells (1×10^7^) were left un-treated or treated with SeV for 8 h and fractionated with differential centrifugation, the P5K and P50K fractions were subjected to WB with the indicated antibodies. Bar graphs represent means ± SD, **P < 0.01, ***P < 0.001, compared with control group.

The role of GP73 in the regulation of IRF3 and NF-*κ*B was evaluated in HEK293 cells co-transfected with plasmids encoding GP73, components of IFN pathway, and ISRE-Luc or NF-*κ*B-Luc. ISRE activities induced by RIG-I, MDA5, MAVS and TBK1 were repressed by GP73, but ISRE activation mediated by IRF3 was not affected by GP73 (Fig 3A, left panel); and NF-*κ*B-Luc activities mediated by TRAF6 and TAK1 were attenuated by GP73, but NF-*κ*B-Luc activation induced by p65/p50 was not affected by GP73 (Fig 3A, right panel); suggesting that GP73 represses IFN signaling downstream of RIG-I/MDA5 and upstream of IRF3/NF-*κ*B.

We then determined whether GP73 is associated with components of IFN pathway in HEK293 cells co-transfected with HA-GP73 and Flag-tagged signaling components. Co-IP revealed that GP73 interacted with MAVS and TRAF6, but not with RIG-I, TRAF3, TBK1 or IRF3 (Fig 3B, top panel). An additional faster band of GP73 was detected in the presence of MAVS or TRAF6 (Fig 3B, bottom panel). We thus determined which band of GP73 was associated with MAVS/TRAF6 in HEK293 cells co-transfected with plasmids expressing GP73 containing both N-terminal HA-tag and C-terminal c-Myc-tag, MAVS or TRAF6. The results demonstrated that the faster band of GP73 containing both the N-terminal HA-tag and the C-terminal c-Myc-tag interacted with MAVS (Fig 3C, left panel) and TRAF6 (Fig 3C, right panel). It was reported GP73 is a glycosylated Golgi-localized protein, depending on the TMD with a positively charged residue in the cytoplasmic N-terminal tail [17, 28]. *In vitro* endoglycosidase digestion assay indicated the faster band of GP73 was lack of glycosylation (S3A Fig). Endogenous CoIP also demonstrated GP73 interacted with MAVS during SeV infection (Fig 3D) or HCV infection (Fig 3E). Further GST pull-down assays (Fig 3F) and confocal microscopy analyses (Fig 3G), indicated that GP73 interacted and co-localized with MAVS/TRAF6. Because the mitochondrial associated membrane (MAM) is the major site of MAVS-mediated IFN signaling [13, 29], while GP73 is a Golgi membrane protein and thus, we analyzed which sub-cellular compartment is the site for MAVS and GP73 interaction. Differential centrifugation and WB demonstrated that GP73 was co-localized with MAVS to the Mito/MAM fraction (P5K) upon SeV infection (Fig 3H). CoIP results of MAVS with Golgi-defective GP73 mutants (WT2-K2E and WT2-ΔK2) also indicated the GP73/MAVS interaction was not dependent on its Golgi localization (S3B Fig).

### TRAF-interacting-motifs are required for the interaction of MAVS with GP73

**Fig 4:**
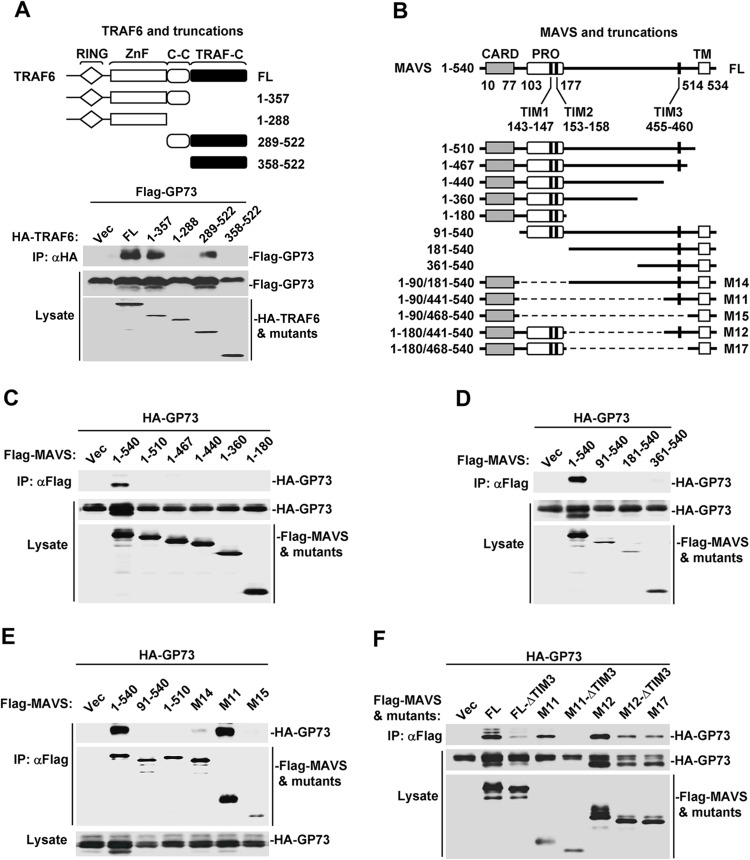
TRAF-interacting-motifs are required for the interaction of MAVS with GP73. (**A**) Scheme of TRAF6 protein and its mutants (top panel). HEK293 cells (2×10^6^) were co-transfected with *GP73* (1 μg) and indicated plasmids (3 μg) for 24 h. Cell lysates were subjected to immunoprecipitation and WB with indicated antibodies. (**B**) Scheme of MAVS and its mutants. (**C**, **D**, **E** and **F**) Interactions of GP73 with MAVS and a series of truncations (C, D and E) or a series of TIM3 mutants (F). HEK293 cells (2×10^6^) were co-transfected with *GP73* plasmid (1 μg) and indicated plasmids (3 μg) for 24 h. Cell lysates were subjected to immunoprecipitation and WB with indicated antibodies.

Since GP73 binds with MAVS/TRAF6, we determined which domain of MAVS/TRAF6 is required for such interaction. Initially, we constructed plasmids expressing TRAF6 and four deletion mutants (Fig 4A, top panel) as described previously [30]. In co-transfected HEK293 cells, GP73 interacted with FL TRAF6, TRAF6-aa 1–357 and TRAF6-aa 289–522, but failed to bind to TRAF6-aa 1–288 or TRAF6-aa 358–522 (Fig 4A, bottom panel), indicating that GP73 binds to coiled-coil domain of TRAF6.

As a central adaptor of IFN signaling, MAVS contains an N-terminal CARD domain required for RIG-I-mediated oligomerization, a proline-rich domain involved in protein-protein interaction, and a C-terminal transmembrane domain (TM) inserting MAVS into mitochondrial outer membrane [4, 31]. MAVS also carries three TRAF-interacting motifs (TIMs) required for the binding of TRAF2/3/5/6 to activate downstream signaling [8, 9, 31]. To determine the functions of MAVS domains in the binding with GP73, we generated a series of MAVS truncations and deletions (Fig 4B). In co-transfected HEK293 cells, GP73 only interacted with FL MAVS (1–540), but not with C-terminal deletions MAVS (1–510), MAVS (1–467), MAVS (1–440), MAVS (1–360) or MAVS (1–180) (Fig 4C), indicating that C-terminal TM domain is required for the interaction with GP73. Similarly, GP73 only interacted with MAVS (1–540), but not with N-terminal deletions MAVS (91–540), MAVS (181–540) or MAVS (361–540) (Fig 4D), suggesting that N-terminal CARD domain is involved in the interaction with GP73. Moreover, GP73 was interacted with MAVS (1–540) and M11 MAVS (1–90/441–540), but not with MAVS (91–540), MAVS (1–510) or M15 MAVS (1–90/468–540) (Fig 4E). These results indicated that CARD domain, TM domain and TIM3 domain are sufficient and efficient for the binding with GP73. Although M14 MAVS (1–90/181–540) contains M11 MAVS and middle sequence of MAVS, it is less efficient in the binding with GP73, which can be explained with an auto-inhibitory effect of middle sequence of MAVS [32]. Interestingly, M15 MAVS (compared with M11, lacking TIM3 and surrounding 20 aa) failed to bind to GP73, which highlighted the importance of TIM3 in MAVS and GP73 interaction. Further analysis of mutations in TIM3 (ΔTIM3: AAANEY instead of PEENEY), that lost the ability of TIM3 to bind TRAF6 [8, 31], showed that GP73 strongly interacted with MAVS (1–540), M11 MAVS (1–90/441–540) and M12 MAVS (1–180/441–540), weakly interacted with FL MAVS ΔTIM3, M12 MAVS ΔTIM3 and M17 MAVS (1–180/468–540), but not with M11 MAVS ΔTIM3 (Fig 4F). *In vitro* GST pull-down assays showed GP73 can directly bind M11 and T6CC (TRAF6 coiled-coil domain) (S4A Fig). To analysis whether putative polyubiquitination of MAVS mediated by activated TRAF6 is necessary in MAVS and GP73 interaction, we made a series of lysine-to-arginine (K to R) point mutations on M11, which contains only four lysine residues. CoIP results showed the M11-KO mutant still interacted with GP73, suggesting polyubiquitination is not necessary for MAVS and GP73 interaction (S4B Fig). Taken together, these results demonstrated that TIMs (TIM1, TIM2 and TIM3) are required for MAVS to interact with GP73, probably through activated TRAF6.

### GP73 facilitates proteasome-dependent degradation of MAVS and TRAF6

**Fig 5:**
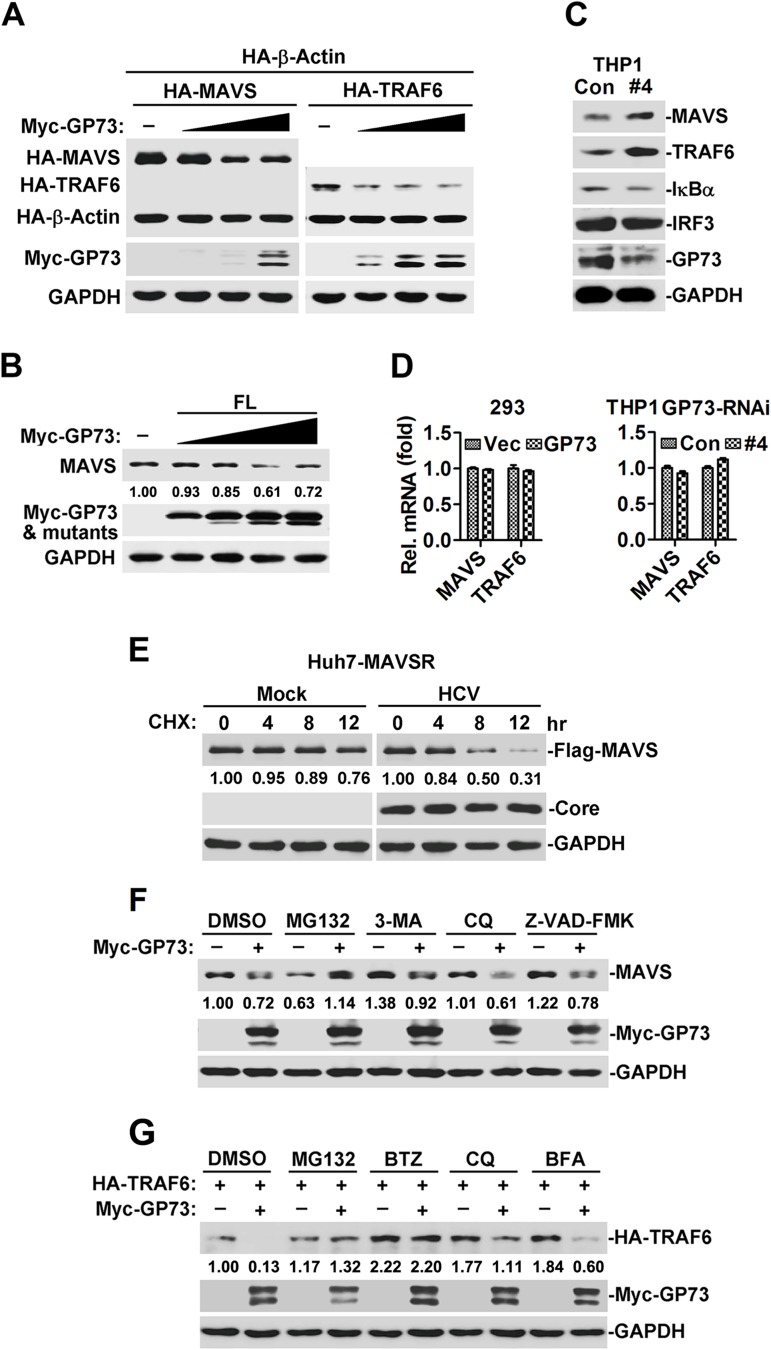
GP73 facilitates proteasome-dependent degradation of MAVS and TRAF6. (**A**) HEK293 cells (2×10^5^) were co-transfected with *GP73* plasmids at 0, 0.125, 0.25 or 0.5 μg and *β-actin* plasmid (0.05 μg) along with *MAVS* or *TRAF6* plasmids (0.5 μg) for 24 h. WCLs were subjected to WB with indicated antibodies. (**B**) HEK293 cells (5×10^5^) were co-transfected with *GP73* plasmids at 0, 0.5, 1, 1.5 or 2 μg for 24 h. Endogenous MAVS was determined by WB. (**C**) THP-1-GP73-RNAi cells were collected and endogenous MAVS, TRAF6, IRF3 and IκBα proteins were detected by WB. (**D**) HEK293 cells (5×10^5^) were transfected with control or *GP73* plasmids for 24 h, the mRNA levels of *MAVS* and *TRAF6* were determined by RT-PCR (left panel). THP-1-GP73-RNAi cells were collected to determine the mRNA levels of *MAVS* and *TRAF6* by RT-PCR (right panel). (**E**) Huh7-MAVSR cells were un-infected or infected with HCV (MOI = 2) for 4 days, CHX (cycloheximide, 40 μg/ml) were added for the indicated time points followed by WB analysis. (**F**) HEK293 cells (2×10^5^) were transfected with *GP73* plasmid (1 μg) for 20 h followed by treatment with DMSO, proteasome inhibitor MG132 (20 μM), autophagosome inhibitor 3-MA (1 mg/ml), lysosome inhibitor CQ (Chloroquine, 100 μM) or apoptosis inhibitor Z-FAD-FMK (50 μM) for 4 h. Endogenous MAVS was detected by WB. (**G**) HEK293 cells (2×10^5^) were co-transfected with *GP73* plasmid (0.1 μg) together with *TRAF6* plasmid (0.2 μg) for 20 h followed by treatment with DMSO, proteasome inhibitor MG132 (20 μM) or BTZ (Bortezomib, 10 μM), lysosome inhibitor CQ (100 μM) or ER-to-Golgi transport inhibitor BFA (Brefeldin A, 20 μg/ml) for 4 h. WCLs were subjected to immunoblots with the indicated antibodies.

Since GP73 interacts with MAVS/TRAF6, we determined whether GP73 affects the stability of MAVS/TRAF6. In HEK293 cells, MAVS and TRAF6 proteins were reduced in the presence of GP73 (Fig 5A), whereas TRAF3 and STING proteins were not affected by GP73 (S5 Fig). In addition, endogenous MAVS protein was attenuated by GP73 over-expression (Fig 5B). Furthermore, endogenous MAVS and TRAF6, but not IRF3 or IκBα, were up-regulated by GP73-shRNA#4 (Fig 5C). These results suggested that over-expression of GP73 down-regulates MAVS/TRAF6, whereas knock-down of GP73 up-regulates MAVS/TRAF6. Interestingly, *MAVS* and *TRAF6* mRNAs were relatively unaffected by over-expression of GP73 (Fig 5D, left panel) or knock-down of GP73 (Fig 5D, right panel), indicating that GP73 does not regulate transcription of *MAVS/TRAF6*. We speculated that GP73 attenuates MAVS/TRAF6 at post-transcriptional level, probably by promoting MAVS/TRAF6 degradation. Moreover, CHX chase results indicated HCV infection promoted MAVS degradation in Huh7-MAVSR cells (Fig 5E).

There are at least three main systems for protein degradation: ubiquitin-proteasome, autophagosome and lysosome pathways. We evaluated which pathway is involved in GP73-mediated MAVS/TRAF6 degradation. MAVS protein was attenuated by GP73, and the degradation of MAVS was diminished by MG132 (proteasome inhibitor), but not by 3-MA (autophagosome inhibitor), CQ (lysosome inhibitor) or Z-VAD-FMK (apoptosis inhibitor) (Fig 5F). Similarly, TRAF6 protein was reduced by GP73, and such reduction was blocked by MG132 and BTZ (proteasome inhibitors), but not by CQ (lysosome inhibitor) or BFA (an inhibitor that disrupts the ER-to-Golgi trafficking of GP73) [27, 33] (Fig 5G). Thus, GP73 promotes MAVS/TRAF6 degradation through proteasome-dependent pathway.

### GP73 facilitates HCV infection and replication

**Fig 6:**
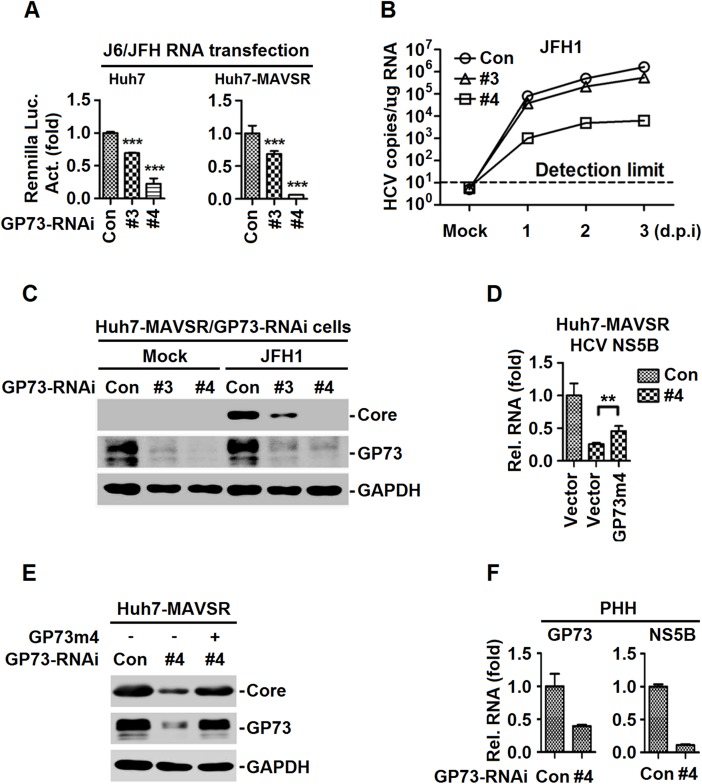
GP73 facilitates HCV infection and replication. (**A**) *GP73*-RNAi-transduced stable Huh7 or Huh7-MAVSR cells were transfected with *in vitro* transcribed FL-J6/JFH5’C19Rluc2Aubi RNA (J6/JFH) for 48 h, and *Renilla* luciferase activities were measured. (**B**, **C**) *GP73*-RNAi-transduced stable Huh7-MAVSR cells were infected with HCV at MOI = 2 for indicated times. Intracellular HCV RNA abundance was determined by qRT-PCR (B), HCV core protein at 3 days post infection (d.p.i) was detected by WB (C). (**D**, **E**) Huh7-MAVSR-GP73-RNAi cells were transfected with *GP73*-rescue plasmid (*GP73m4*) for 24 h, followed by HCV infection at MOI = 2 for 3 days. HCV RNAs were determined by RT-PCR (D) and HCV core protein was detected by WB (E). (**F**) PHHs were transduced with *GP73*-shRNA lentivirus for 48 h, followed by HCV infection at MOI = 2 for 2 days. HCV RNA levels were determined by RT-PCR. Bar graphs represent means ± SD, **P < 0.01 compared with control group.

Since HCV activates GP73 in immune-competent cells (PHHs and Huh7-MAVSR) and GP73 promotes MAVS/TRAF6 degradation, leading to the attenuation of IFN signaling, we determined the function of GP73 in the regulation of HCV infection and replication. Huh7 and Huh7-MAVSR cells stably expressing *GP73*-shRNAs were transfected with *in vitro* transcribed J6/JFH-Rluc HCV genomic RNA. HCV infection (indicated by Renilla luciferase activity) (Fig 6A), HCV genomic RNA replication (indicated by HCV RNA copy) (Fig 6B) and HCV core protein production (Fig 6C) were attenuated by *GP73*-shRNA#3 and *GP73*-shRNA#4. These results demonstrated that knock-down of *GP73* down-regulates HCV infection, viral RNA replication and protein production.

In addition, the role of *GP73*-shRNA#4 in HCV replication was evaluated by rescue experiments. A *GP73*-rescue construct (*GP73m4*) that is resistant to *GP73*-shRNA#4 targeting without changing the sequence of GP73 was generated. HCV RNA expression (Fig 6D) and core protein production (Fig 6E) were repressed by *GP73*-shRNA#4, and such repressions were rescued by GP73m4. We further showed that lentivirus transduced GP73m4 fully rescued the HCV RNA and core protein production, repressed by *GP73*-shRNA#4 (S6A and S6B Fig). Moreover, the effect of GP73 on HCV replication was determined in PHHs transduced with lentivirus-*GP73*-shRNA#4 and infected with HCV. HCV RNA was repressed by *GP73*-shRNA#4 (Fig 6F), indicating knock-down of GP73 down-regulates HCV replication. Finally, we evaluated the effect of GP73 on the replication of VSV in VSV-*GFP*-infected Huh7-*GP73*-shRNAs cells. VSV infection was attenuated by *GP73*-shRNA#3 and repressed by *GP73*-shRNA#4 (S6C Fig), suggesting that GP73 plays a stimulatory role in VSV replication. Taken together, GP73 facilitates HCV infection, viral RNA replication and protein production.

## Discussion

**Fig 7:**
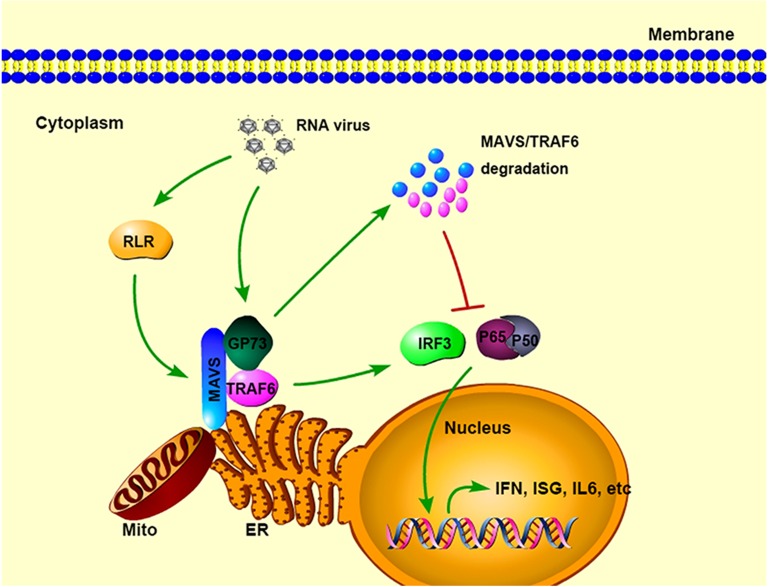
A proposed model for the role of GP73 in HCV triggered IFN signaling. During HCV infection, GP73 expression is activated and correlated with IFN-β production. Subsequently, GP73 binds MAVS and TRAF6 to facilitate MAVS and TRAF6 proteasome-dependent degradation, leading to the repression of host innate immunity and facilitation of HCV infection.

We discovered a key function of GP73 in the regulation of host innate immunity and revealed a novel mechanism by which GP73 regulates HCV replication (Fig 7). Initially, we showed that HCV infection activates GP73 in patients’ serum, PHHs and human hepatoma cells. Subsequently, we demonstrated that GP73 in turn binds directly with MAVS and TRAF6 to promote MAVS/TRAF6 degradation, which result in the repression of host innate immunity and facilitation of HCV infection.

It is known that the mitochondrial-associated ER membrane (MAM) is the major site of MAVS signaling, and HCV NS3/4A protease cleaves MAVS synapse from MAM, but not from mitochondria, to ablate immune defenses [29]. Upon RIG-I/MDA5 activation, MAVS forms large prion-like aggregates to propagate antiviral innate immunity by recruiting TRAF2/3/6 E3 ubiquitin ligases [34]. ER-to-Golgi transport compartments mediate the dynamic association of TRAF3 with MAVS at MAM, leading to inducing innate immune responses [35]. Our studies showed that GP73, a resident Golgi membrane protein, is recruited to MAVS/TRAF6 signalosome on MAM to inhibit immune defenses against HCV infection, which highlight the roles of Golgi apparatus and membrane compartments traffic in modulating innate immunity. Proteomic analysis during RNA virus infection also indicates GP73 traffic to MAM during RNA virus infection [36]. Knock-down of *GP73* in Huh7-MAVSR cells transfected with *in vitro* transcribed HCV genomic RNA activates IFN production, whereas in Huh7 cells transfected with J6/JFH1-Rluc RNA impairs HCV replication. We could not exclude the possibility that GP73 may have other strategies to facilitate HCV infection, such as promoting HCV particle assembly and secretion [24], or mediating MAVS-NS3/4A interaction on MAM. We also reported that GP73 mediates the interaction of ApoE and HCV NS5A to promote viral secretion [37]. Further studies are needed to clarify other functions of GP73 in HCV life cycle and HCV associated liver diseases.

Negative regulation of innate immunity plays an important role in maintaining the balance of cell signaling. The adaptor protein MAVS and the ubiquitin E3 ligase TRAF6 play central roles in virus-triggered innate immunity. We revealed that GP73 interacts with TRAF6/MAVS and the interaction of GP73 with MAVS is dependent on TRAF-interacting-motifs. Because after TRAF6 recruitment by MAVS activation, TRAF6 synthesizes polyubiquitin chains on MAVS followed by the recruitment of TBK1 and IKKβ, leading to MAVS phosphorylation and subsequent IRF3 activation [9]. Possibilities are existed that the polyubiquitination or phosphorylation modifications serve as scaffolds for GP73 binding, just likes the model of TBK1 and IRF3 binding to MAVS [9]. The M11-KO mutant CoIP and *in vitro* GST pull-down experiments further indicated the direct binding of GP73 with MAVS, but not the polyubiquitin chains. TRAF6 is known as an E3 ubiquitin ligase that mediates synthesis of K63-linked polyubiquitin chains, while K48-linked polyubiquitination chains target proteins for proteasome-mediated degradation. As not an E3 ubiquitin ligase, GP73 must recruit E3 ligases to MAVS/TRAF6 signalosome to synthesize K48-linked polyubiquitin chains. The exact E3 ligase involved in GP73-mediated proteasome degradation of MAVS/TRAF6 remains unidentified.

More recently, it reported that single-nucleotide polymorphism (SNP) of *GP73* is associated with cytokine signaling in peripheral blood mononuclear cells (PBMCs) upon bacteria and fungi stimulation [38]. This study further expands that GP73 acts as a negative regulator of innate immunity to facilitate HCV infection by interacting with MAVS/TRAF6 to promote MAVS/TRAF6 degradation. We also showed that GP73 plays a stimulatory role in VSV replication. Taken together, these results suggested that GP73 plays a broad role in the regulation of pathogen infection, including fungi, bacteria and viruses. This study provides new insights into the mechanism of HCV infection and pathogenesis, and also suggests that GP73 acts as a potential antiviral target in the prevention and treatment of pathogen infections.

## Materials and methods

### Human serum specimens

Participants included 60 HCV patients randomly retrieved from Renmin Hospital of Hubei Province from September 2015 to January 2016. All participants were diagnosed with HCV infection by the presence of anti-HCV antibodies and serum HCV RNA (Table 1), and confirmed as negative for hepatitis B virus (HBV) and human immunodeficiency virus-1 (HIV-1). Serum samples from healthy individuals were randomly selected from a local blood donation center.

### Ethics statement

The study was conducted according to the principles of the Declaration of Helsinki and approved by the Institutional Review Board of the College of Life Sciences, Wuhan University, in accordance with its guidelines for the protection of human subjects. All participants have provided written informed consent to participate in the study.

### Cell lines and cultures

Primary human hepatocytes (PHHs) were purchased from Research Institute for Liver Diseases (Shanghai, China) and cultured as described previously [16]. Cells were infected with JFH1 HCVcc at MOI of 2 for indicated times. To study the effect of knock-down of *GP73* on HCV infection in PHHs, plated PHHs were transduced with lentiviruses expressing shRNA targeting *GP73* for 48 h, and then infected with JFH1 HCVcc at MOI of 2 for 2 days followed by RT-PCR analysis.

Human hepatoma cell lines (HepG2 and Huh7, human normal liver cell line (L02), human embryonic kidney HEK cell line (HEK293), human acute monocytic leukemia cell line (THP-1) and human epitheloid cervix carcinoma cell line (HeLa) were purchased from American Type Culture Collection (ATCC) (Manassas, VA, USA) and described previously [39]. Human hepatoma cell line (Huh7.5.1) was kindly provided by Dr. Francis V Chisari of Scripps Research Institute, USA. Huh7-MAVSR cell line was provided by Dr. Jin Zhong of Institute Pasteur, Shanghai, China [16]. THP-1 cells were cultured in RPMI 1640 medium containing 10% heat-inactivated fetal bovine serum (FBS) (Gibco, Grand Island, NY, USA). Other cell lines were cultured in Dulbecco’s modified Eagle’s medium (DMEM) purchased from Gibco (Grand Island, NY, USA) containing 10% FBS. All cell cultures were maintained at 37°C in a 5% CO_2_ incubator.

### Viruses and infection

JFH1 HCVcc supernatant was prepared from Huh7.5.1 cells transfected with *in vitro*-transcribed JFH1 genomic RNA. The amplification, concentration, purification and titration of HCVcc were performed as described previously [40]. Briefly, Huh7.5.1 cells were infected with HCVcc at an MOI of 0.01 for 7–9 days. Cells were sub-cultured once before confluence. The supernatant were collected and concentrated at 28,000 rpm for 4 h at 4°C (SW28 rotor), the pellets were resuspended and loaded to a 20–60% sucrose gradient at 120,000 **×** g for 16 h at 4°C (SW41i rotor). Fractions of 1 ml were collected from the top of gradient and determined by RT-qPCR. High HCV RNA fractions were titrated on Huh7.5.1 cells by immunofluorescence staining against HCV NS5A 3 days postinfection. The infections of recombinant vesicular stomatitis virus-green fluorescent protein (VSV-GFP) and enterovirus 71 (EV71) were as described previously [41]. Briefly, VSV-GFP were produced in HEK293 cells and titrated by counting GFP-positive cell numbers. EV71 were produced in RD cells and titrated by TCID50. SeV were propagated in embryonated eggs and titrated by blood coagulation test.

### Plasmids construction

FL-J6/JFH5’C19Rluc2Aubi was a gift from Dr. Charles M. Rice of Rockefeller University and prepared as described previously [42]. JFH1 HCV replicon was kindly provided by Dr. Takaji Wakita of National Institute of Infectious Diseases, Tokyo and prepared as described previously [43]. *IFN-β-Luc*, *ISRE-Luc* or *NF-κB-Luc* reporter plasmids and HA-tagged full length (FL) TRAF6 and its truncations were gifts from Dr. Ying Zhu of Wuhan University, China and constructed as previously described [30, 41]. Mammalian expression plasmids for HA-, Flag- or c-Myc-tagged *GP73*, *RIG-I*, *MDA5*, *MAVS*, *STING*, *TRAF3*, *TRAF6*, *TBK1*, *IRF3*, *TAK1*, *TAB1*, *p65*, *p50*, *β-actin* and the truncated proteins were constructed by standard molecular cloning method from cDNA templates. Unless otherwise described, GP73 constructs were C-terminal tagged. To generate *GST-GP73* plasmid, GP73 coding sequence lacking the N-terminal transmembrane domain (aa36-401) was sub-cloned into the *Bam*HI/*Xho*I sites of pGEX-6p-1 vector. MBP-M11 and MBP-T6CC were constructed by inserting the M11 or T6CC sequences into the *EcoRI/SalI* sites of pMAL-c2x vector. All constructs were confirmed by DNA sequencing. The following primers were used to generate *GP73*-rescue construct *GP73m4* by site-directed mutagenesis: *M4F*: 5’-GTgGAaAAgGAaGAgACgAAcGAGATCCAGGTGGTGAATGAG-3’; *M4R*: 5’-gTTcGTcTCtTCcTTtTCcACTTGTCTCTTTGAATCCAAAACCAC-3’.

### Antibodies

Antibodies against GP73, MAVS, GAPDH, AIF, MBP and GST were purchased from Proteintech (Wuhan, Hubei, China). Antibody against HCV NS5A (2F6) was purchased from BioFront (Anhui, Hefei, China). Antibodies against HCV Core and p-IRF3 were purchased from Abcam (Cambridge, MA, USA). Antibody against IRF3 was purchased from Santa Cruz Biotechnology (Dallas, Texas, USA). Antibodies against Flag, HA, c-Myc, TRAF6, Calnexin, Syntaxin 6, p-IκBα and IκBα were purchased from Cell Signaling Technology (Danvers, MA, USA). All inhibitors were purchased from Selleck Chemicals (Houston, TX, USA).

### Quantitative real-time PCR analysis

Total RNA was extracted from cells using TRIzol reagent (Invitrogen), then 1 μg of total RNA was used to synthesize cDNA with Moloney murine leukemia virus reverse transcriptase (Promega) and N6 random primer for 1 h at 37°C and subjected to real-time PCR analysis with specific primers. HCV RNA levels were also determined relative to a standard curve composed of serial dilutions of DNA template containing the JFH1 NS5B cDNA. Gene-specific primer sequences were as described previously [7, 16] and listed in S1 Table.

### Gene silencing with lentiviral shRNA system

The short hairpin targeting sequences for specific genes were cloned into the pLKO.1-TRC control vector (A gift from David Root, addgene #10879). Lentivirus were produced in HEK293 cells by co-transfection of pMD2.G (Addgene #12259), psPAX2 (Addgene #12260) and pLKO.1-shRNA plasmids (Ratio 1:3:4). Virus supernatant was collected at 48 and 72 h post transfection, and passed through a 0.45 μm filter, followed by PEG-8000 precipitation, aliquots were stored at -80°C. To establish a stable knockdown cell line, the shRNA lentivirus stocks were used to transduce Huh7 cells or THP-1 cells with 8 μg/ml polybrene. At 48 h post transduction, cells were cultured in puromycin (2.5 μg/ml for Huh7 cells and 1 μg/ml for THP-1 cells) selection medium for at least 7 days. To establish stable knockdown cells in Huh7-MAVSR, we made a neomycin-resistant version of pLKO.1 by replacing the Puro to Neo, and the lentivirus-transduced cells were selected in G418 (500 μg/ml) for 10 days. The following sequences were targeted for human *GP73* CDS: #2: 5’-CCACAGGATTTGAGATGCTAA-3’; #3: 5’-CGAATAGAAGAGGTCACCAAA-3’; #4: 5’-GTTGAGAAAGAGGAAACCAAT-3’. The target sequence of the seed sequence-matched control of #4 were as followed: *S4C*, 5’-GTTGAGAAActcGAAACCAAT-3’.

### Immunoprecipitation

Cells were collected and lysed in IP-lysis buffer (50 mM Tris-HCl, 150 mM NaCl, 1% Triton X-100, 1 mM EDTA, 10% glycerol, and protease inhibitor cocktail, pH7.4). Supernatants were collected by centrifugation (15,000 *g*, 15 min, 4°C), and were pre-cleared with 30 μl protein G-conjugated agarose (GE Healthcare Life Sciences) followed by centrifugation (2,000 *g*, 2 min, 4°C). The pre-cleared supernatants were incubated with the indicated antibodies (1 μg/ml) for 3 h or overnight at 4°C, followed by immunoprecipitation with 30 μl protein G-conjugated agarose for 2 h at 4°C. The precipitates were washed 5–7 times with IP-wash buffer (50 mM Tris-Cl, 300 mM NaCl, 1% Triton X-100, 1 mM EDTA, pH7.4) and detected through WB.

### Immunofluorescence

Cells were fixed with 4% paraformaldehyde for 15 min, followed by permeabilization with 0.5% Triton X-100 for 20 min at room temperature. Primary antibodies (0.2 μg/ml) were added for 2 h at room temperature post blocking with 5% bovine serum albumin for 1 h. Samples were further stained with FITC-, DyLight649-conjugated secondary antibodies, followed by visualization with confocal microscopy (Olympus FV1000).

### Endoglycosidase digestion assay

For an *in vitro* endoglycosidase digestion assay, HEK293 cells (5×10^5^) were co-transfected with HA-*GP73-Myc* (1 μg) and Flag-*MAVS* or Flag-*TRAF6* (1 μg) for 24 h. Cells were lysed in 50 μl IP-lysis buffer, and half lysates were denatured and digested with 500 U Endo H (NEB) for 3 h at 37°C before WB analysis.

### Cell fractionation assays

The cell fractionation assay was performed by differential centrifugation as described [44]. Briefly, HEK293 cells (1×10^7^) were left un-treated or treated with SeV for 8 h and were washed in PBS followed by dousing 40 times in 2 ml homogenization buffer (10 mM Tris-HCl, pH7.4, 2 mM MgCl_2_, 10 mM KCl, and 250 mM sucrose). The homogenate was centrifuged at 500 g for 10 min to pellet un-broken cells and nuclei (twice). The supernatant was centrifuged at 5,000 g for 10 min to precipitate crude mitochondira (P5K, wash once). The supernatant (S5K, pellet twice) was further centrifuged at 50,000 g for 60 min to precipitate membrane fractions (P50K).

### GST pull down

For an *in vitro* binding assay, the GST-fused GP73 or GST alone was expressed in BL21 cells and purified with glutathione Sepharose 4B (GE Healthcare Life Sciences) according to the supplier’s instructions. HEK293 cells transfected with Flag-*MAVS* or Flag-*TRAF6* were washed twice with PBS and lysed in GST-lysis buffer (20 mM Tris-Cl, 200 mM NaCl, 1 mM EDTA, 0.5% NP-40 and protease inhibitor cocktail, pH7.5). Supernatants were subjected to GST pull down with 10 μg of GST or 20 μg of GST-GP73 overnight at 4°C. After being washed with GST-lysis buffer for four times, proteins were extracted from the Sepharose beads by boiling in 2× SDS loading buffer, and detected through WB. The MBP-fused M11, T6CC or MBP alone was expressed in BL21 cells and purified with amylose resin (NEB) according to the supplier’s instructions. For an *in vitro* binding assay, purified MBP, MBP-M11 or MBP-T6CC (20 μg) were subjected to GST pull-down with 10 μg of GST or 20 μg of GST-GP73 overnight at 4°C as described above.

### Transfection and reporter assays

HEK293 cells were plated and transfected the following day by lipofectamine 2,000 (Invitrogen). Empty control plasmids were added to ensure that each transfection receives the same amount of total DNA. Luciferase reporter vectors were co-transfected with pRL-TK (as an internal control reporter vector) into HEK293 cells. Dual luciferase assays were performed with guidelines provided by the manufacturer (Promega).

### ELISA

Serum samples of healthy individuals or HCV patients were collected and stored at -80°C. Supernatants from cultured cells were collected at the indicated time points. The GP73 protein levels were analyzed by ELISA kits with the manufacturer’s instructions (Hotgen Biotech, China).

### Statistical analysis

Statistical graphs were created with Origin or GraphPad Prism software, and statistical analysis was performed with two-tailed Student’s *t* tests. The graphs represent the mean values ± the standard deviations (SD) of at least three independent experiments. *P* < 0.05 was considered statistically significant, and *P* < 0.01 and *P* < 0.001 were considered highly significant.

## Supporting information

S1 Fig GP73 expression is activated and correlated with IFN activation during virus infection. (**A**, **B**) HEK293 cells were infected VSV-*GFP* at an MOI of 0.1 or 0.2 for 12 h (A). SK-N-SH cells were infected with EV71 at an MOI of 1 or 2 for 12 h. The mRNA levels of *GP73* and *IFN-β* were determined through RT-PCR (B). (**C**) Huh7 cells were treated with IFN-β (300 unit/ml) or IFN-*λ*1 (20 ng/ml) for 8 h, the mRNA level of *GP73* and *MxA* were determined through RT-PCR. (**D**) Huh7 cells were treated with indicated inhibitors for 24 h. *GP73* mRNAs were determined by RT-PCR. (**E**) Huh7 cells were treated with U0126 or PD98059 at different concentrations as indicated for 24 h, followed by RT-PCR analysis. (**F**, **G**) Huh7 cells were treated with increasing concentration of Rapamycin at concentrations as indicated for 24 h, followed by RT-PCR analysis (F) and WB detection (G).
(TIF)

S2 Fig GP73 represses host innate immunity during viral infection. (**A**) The scheme of GP73 conserved domains and truncations as reported. (**B**) HEK293 cells (1×10^5^) were co-transfected with *IFN-β* reporter plasmid (0.1 μg) and a series of *GP73* truncation plasmids (0.2 μg) for 24 h, and then infected with SeV for 10 h before luciferase reporter assays were performed. (**C**) HEK293 cells (2×10^5^) were transfected with a series of *GP73* truncation plasmids (0.5 μg) for 24 h, the expression of GP73 truncations were detected by WB. (**D**) The effects of knock-down of *GP73* on the expression of *GP73* mRNA and GP73 protein. HEK293 cells were transiently transfected with the control (Con) or *GP73*-shRNAs plasmids as indicated for 36 h. GP73 expression was determined by RT-PCR and WB. (**E**) The expression status of *GP73* in different cell lines. The relative mRNA levels of *GP73* in different cell lines were determined by RT-PCR. (**F**) *GP73*-shRNA#4 or its seed sequence-matched control S4C transduced stable Huh7 cells were infected with SeV for 12 h. *IFN-β*, *IFN-λl*, *IL-6* and *ISG56* mNRAs were quantified by RT-PCR. Bar graphs represent means ± SD, *P < 0.05, **P < 0.01, ***P < 0.001, compared with control group.
(TIF)

S3 Fig MAVS and TRAF6 interact with the faster band of GP73. (**A**) HEK293 cells (5×10^5^) were co-transfected with HA-*GP73-Myc* (1 μg) and Flag-*MAVS* or Flag-*TRAF6* (1 μg) for 24 h. Cells were lysed and lysates were denatured and digested with 500 U Endo H for 3 h at 37°C before WB analysis. (**B**) HEK293 cells (2×10^6^) were co-transfected with Flag-*MAVS* (2 μg) and Myc-tagged *GP73* or mutants (3 μg) for 24 h. Cells were lysed and lysates were immunoprecipitated with anti-Myc. Immunoprecipitates and WCLs were analyzed by WB with indicated antibodies.
(TIF)

S4 Fig GP73 directly binds MAVS and TRAF6. (**A**) The purified recombinant MBP-lacZα (Vec) or MBP-M11 or MBP-T6CC (TRAF6 coiled-coil domain) (20 μg) were subjected to GST pull down assays with equal molar quantity of purified GST (10 μg) or recombinant GST-GP73 (20 μg) proteins. Immunoblots were performed with indicated antibodies. (**B**) HEK293 cells (2×10^6^) were co-transfected with Flag-tagged *MAVS FL* or *M11* or mutants (3 μg) together with HA-*GP73* or mutants (1 μg) for 24 h. Cells were lysed and lysates were immunoprecipitated with anti-Flag. Immunoprecipitates and WCLs were analyzed by WB with indicated antibodies.
(TIF)

S5 Fig The effect of GP73 on the expression of co-transfected TRAF3 and STING. HEK293 cells (2×10^5^) were transfected with control plasmid or plasmids expressing *GP73* at different concentrations as indicated (0, 0.125, 0.25 or 0.5 μg), *β-actin* (0.05 μg), and *TRAF3* (0.5 μg) or *STING* (0.5 μg) for 24 h. Whole cell lysates were subjected to WB with the indicated antibodies.
(TIF)

S6 Fig GP73 facilitates HCV and VSV infection. (**A, B**) Huh7-*MAVSR*-*GP73*-RNAi cells were transduced with lentivirus-Vec or lentivirus-*GP73m4* for 48 h, followed by HCV infection at MOI = 2 for 3 days. HCV RNAs were determined by RT-PCR (A) and HCV core protein was detected by WB (B). (**C**) The Huh7-GP73-RNAi cells were plated and infected with VSV-*GFP* (MOI = 1) for 12 h, followed by analyzing and counting the GFP-positive cells under a fluorescence microscope. ***p < 0.001 compared with control group.
(TIF)

S1 Table Primers used in this study. (DOC)
