# Correction: GP73 represses host innate immune response to promote virus replication by facilitating MAVS and TRAF6 degradation

**DOI:** 10.1371/journal.ppat.1006938

**Published:** 2018-03-13

**Authors:** Xuewu Zhang, Chengliang Zhu, Tianci Wang, Hui Jiang, Yahui Ren, Qi Zhang, Kailang Wu, Fang Liu, Yingle Liu, Jianguo Wu

The following information is missing from the Funding section: This work was supported by grants from National Natural Science Foundation of China (81730061, 31230005, 81471942, 31200134, and 31270206), and the National Mega Project on Major Infectious Disease Prevention (2017ZX10103005). The funders had no role in study design, data collection and analysis, decision to publish, or preparation of the manuscript.
