# Supplementary figures and images for: GP73 represses host innate immune response to promote virus replication by facilitating MAVS and TRAF6 degradation
Source: PLoS Pathog. 2017 Apr 10;13(4):e1006321. doi: 10.1371/journal.ppat.1006321 (PMC5398727; doi:10.1371/journal.ppat.1006321)

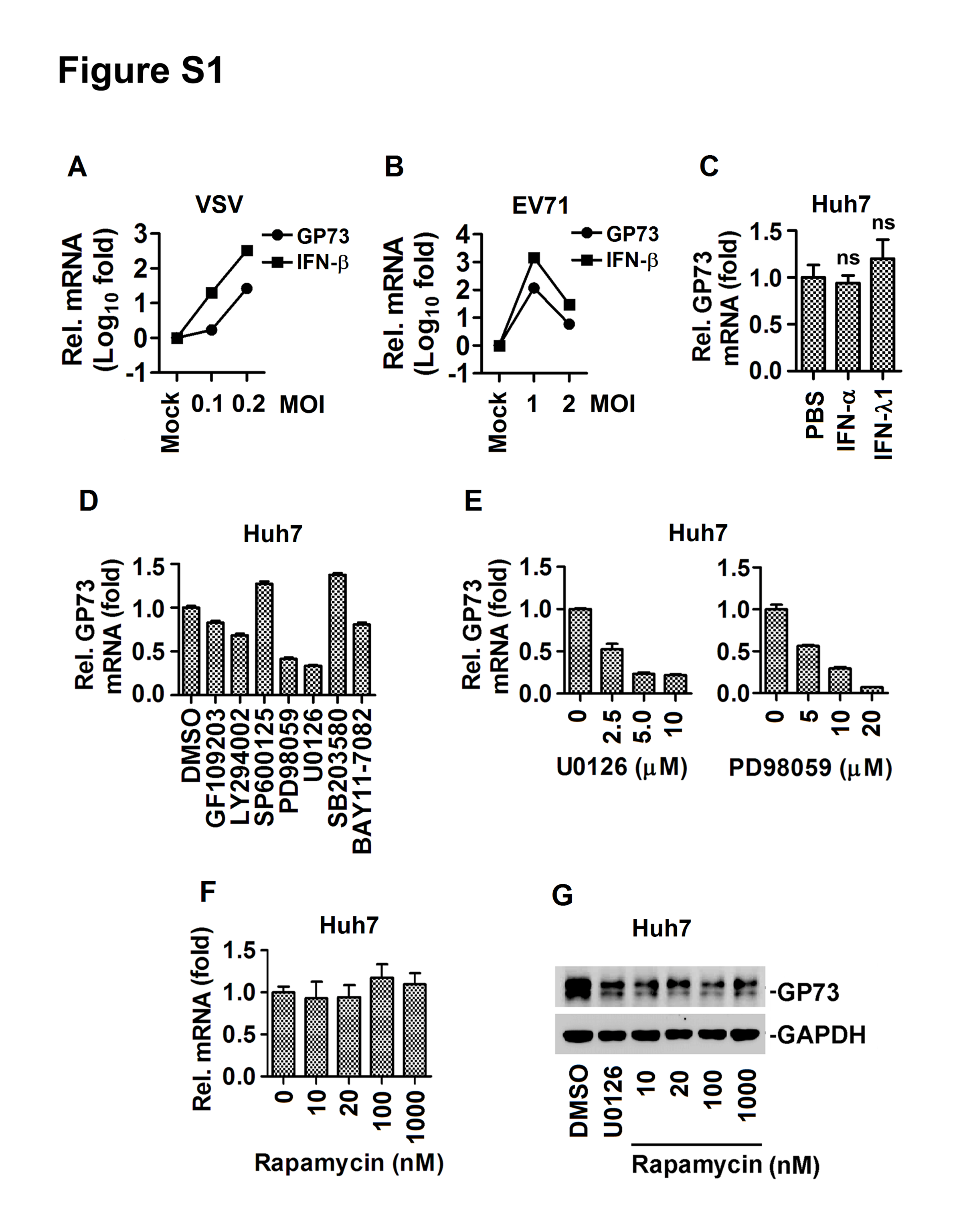

Supplement: S1 Fig — (A, B) HEK293 cells were infected VSV-GFP at an MOI of 0.1 or 0.2 for 12 h (A). SK-N-SH cells were infected with EV71 at an MOI of 1 or 2 for 12 h. The mRNA levels of GP73 and IFN-β were determined through RT-PCR (B). (C) Huh7 cells were treated with IFN-β (300 unit/ml) or IFN-λ1 (20 ng/ml) for 8 h, the mRNA level of GP73 and MxA were determined through RT-PCR. (D) Huh7 cells were treated with indicated inhibitors for 24 h. GP73 mRNAs were determined by RT-PCR. (E) Huh7 cells were treated with U0126 or PD98059 at different concentrations as indicated for 24 h, followed by RT-PCR analysis. (F, G) Huh7 cells were treated with increasing concentration of Rapamycin at concentrations as indicated for 24 h, followed by RT-PCR analysis (F) and WB detection (G). (TIF) [file ppat.1006321.s001.tif]

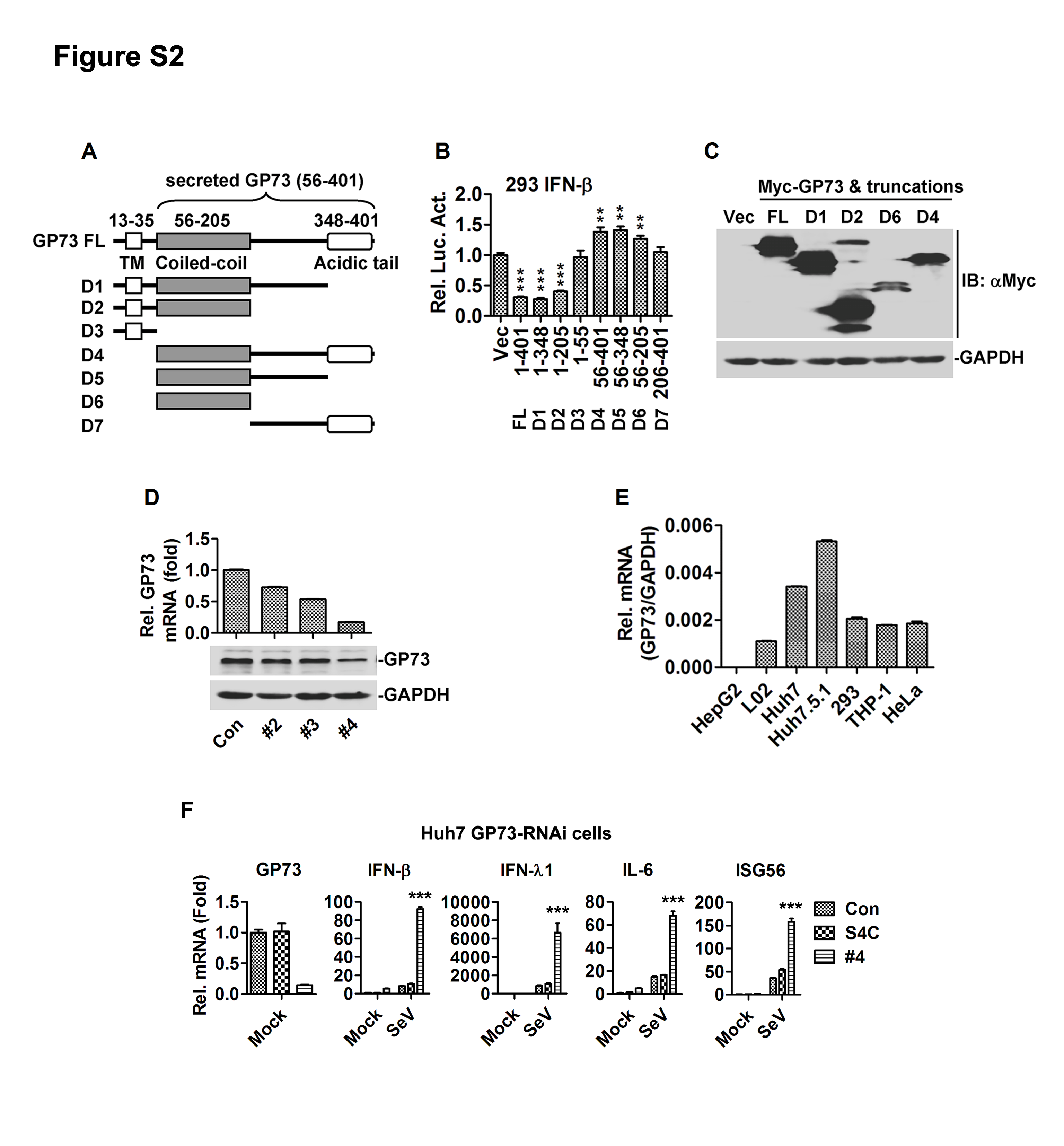

Supplement: S2 Fig — (A) The scheme of GP73 conserved domains and truncations as reported. (B) HEK293 cells (1×10^5^) were co-transfected with IFN-β reporter plasmid (0.1 μg) and a series of GP73 truncation plasmids (0.2 μg) for 24 h, and then infected with SeV for 10 h before luciferase reporter assays were performed. (C) HEK293 cells (2×10^5^) were transfected with a series of GP73 truncation plasmids (0.5 μg) for 24 h, the expression of GP73 truncations were detected by WB. (D) The effects of knock-down of GP73 on the expression of GP73 mRNA and GP73 protein. HEK293 cells were transiently transfected with the control (Con) or GP73-shRNAs plasmids as indicated for 36 h. GP73 expression was determined by RT-PCR and WB. (E) The expression status of GP73 in different cell lines. The relative mRNA levels of GP73 in different cell lines were determined by RT-PCR. (F) GP73-shRNA#4 or its seed sequence-matched control S4C transduced stable Huh7 cells were infected with SeV for 12 h. IFN-β, IFN-λl, IL-6 and ISG56 mNRAs were quantified by RT-PCR. Bar graphs represent means ± SD, *P < 0.05, **P < 0.01, ***P < 0.001, compared with control group. (TIF) [file ppat.1006321.s002.tif]

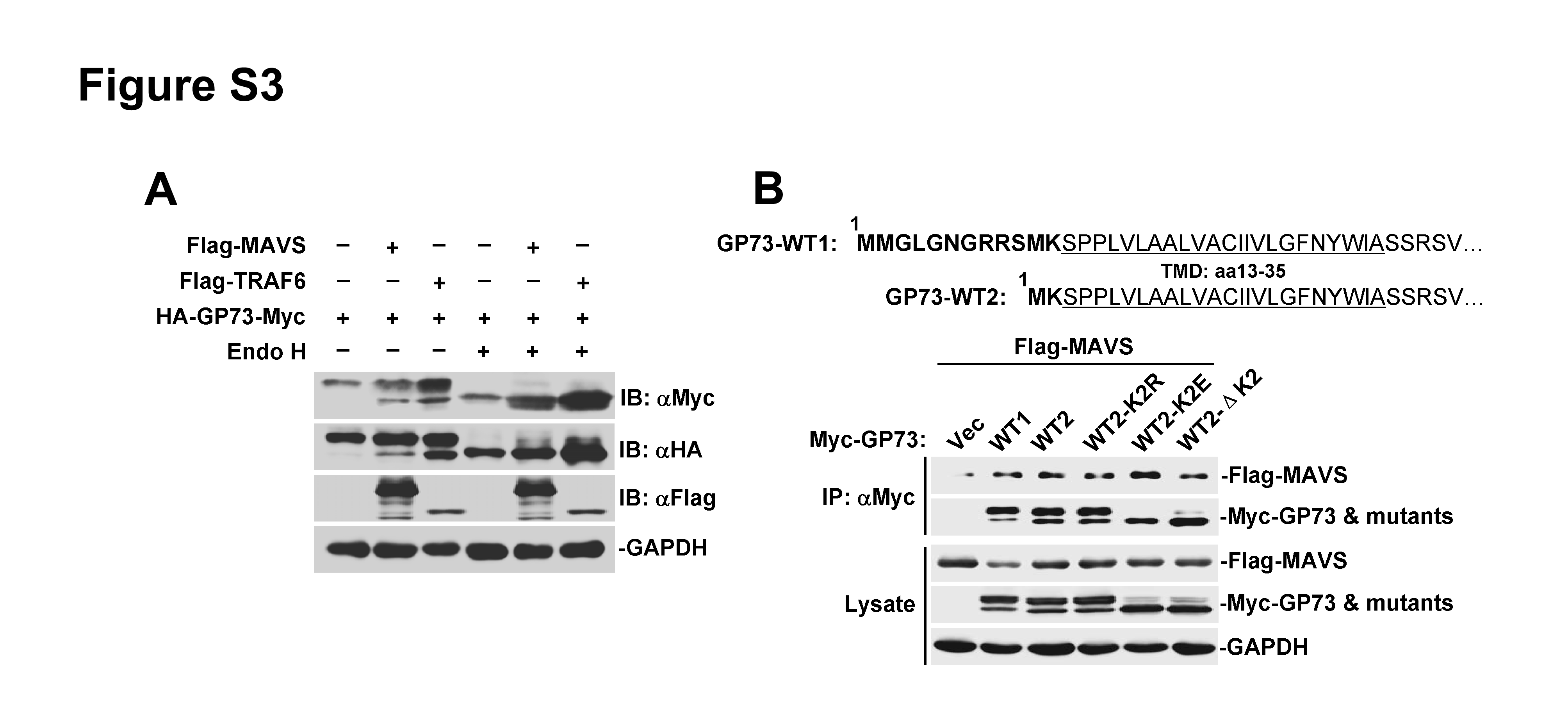

Supplement: S3 Fig — (A) HEK293 cells (5×10^5^) were co-transfected with HA-GP73-Myc (1 μg) and Flag-MAVS or Flag-TRAF6 (1 μg) for 24 h. Cells were lysed and lysates were denatured and digested with 500 U Endo H for 3 h at 37°C before WB analysis. (B) HEK293 cells (2×10^6^) were co-transfected with Flag-MAVS (2 μg) and Myc-tagged GP73 or mutants (3 μg) for 24 h. Cells were lysed and lysates were immunoprecipitated with anti-Myc. Immunoprecipitates and WCLs were analyzed by WB with indicated antibodies. (TIF) [file ppat.1006321.s003.tif]

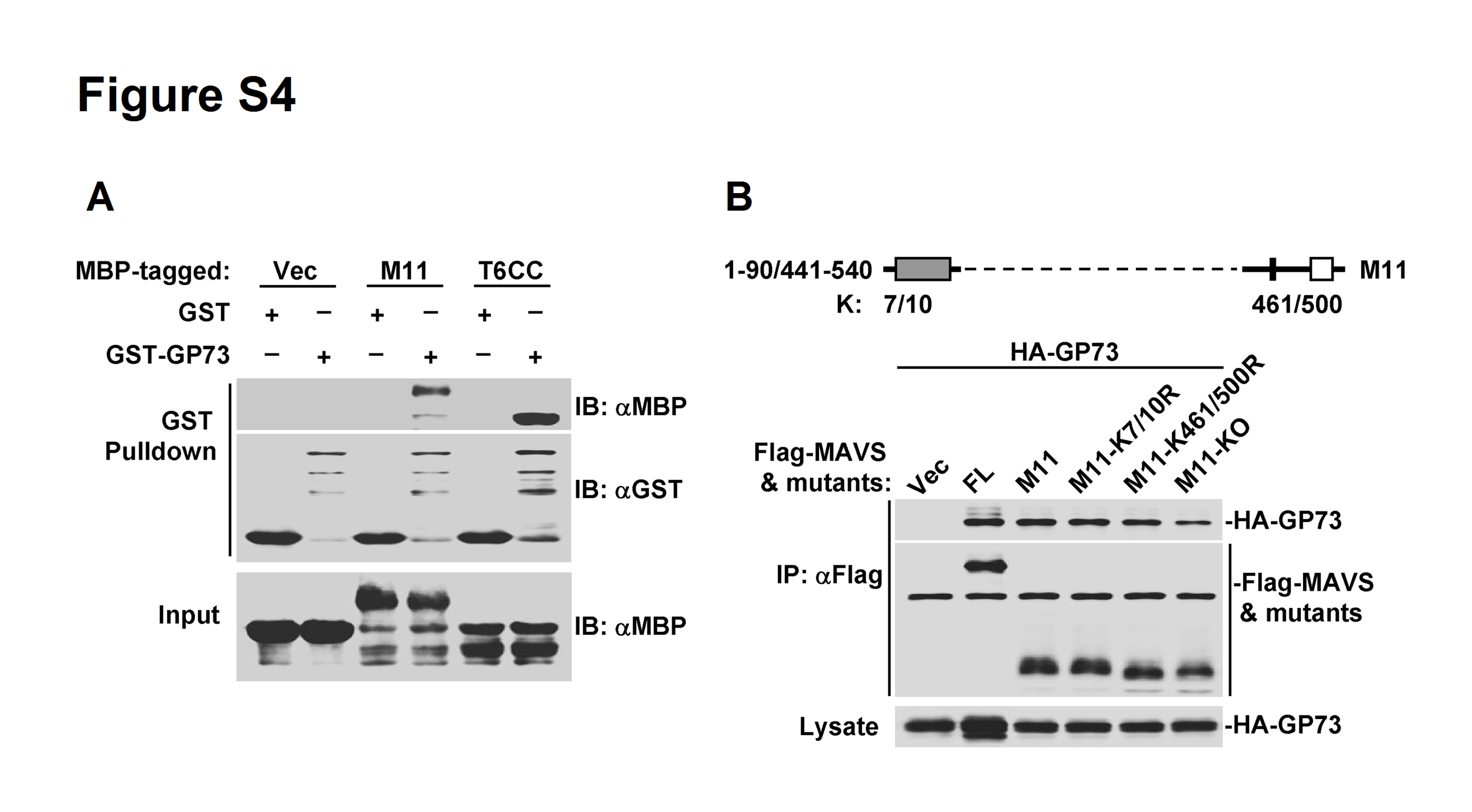

Supplement: S4 Fig — (A) The purified recombinant MBP-lacZα (Vec) or MBP-M11 or MBP-T6CC (TRAF6 coiled-coil domain) (20 μg) were subjected to GST pull down assays with equal molar quantity of purified GST (10 μg) or recombinant GST-GP73 (20 μg) proteins. Immunoblots were performed with indicated antibodies. (B) HEK293 cells (2×10^6^) were co-transfected with Flag-tagged MAVS FL or M11 or mutants (3 μg) together with HA-GP73 or mutants (1 μg) for 24 h. Cells were lysed and lysates were immunoprecipitated with anti-Flag. Immunoprecipitates and WCLs were analyzed by WB with indicated antibodies. (TIF) [file ppat.1006321.s004.tif]

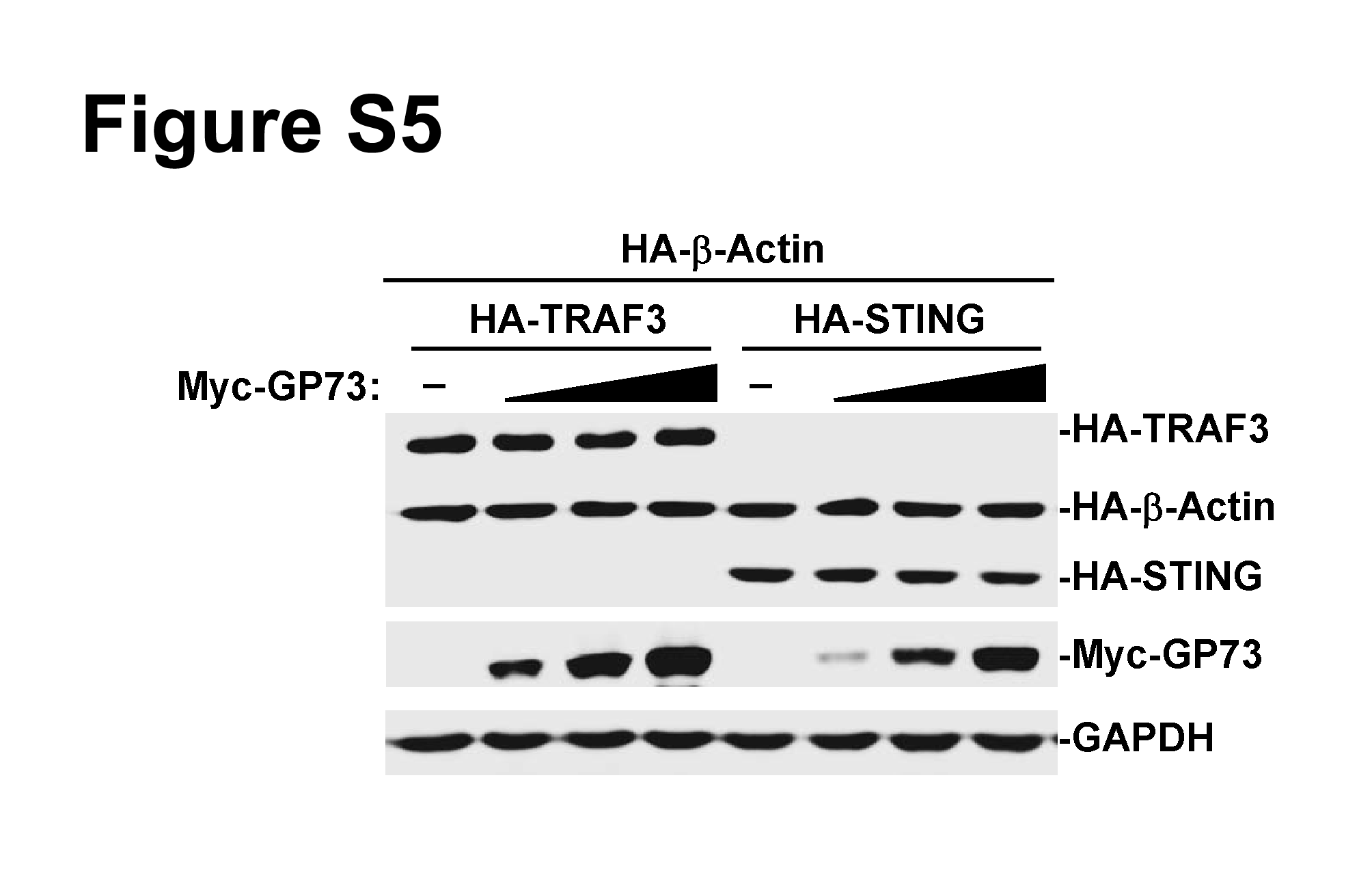

Supplement: S5 Fig — HEK293 cells (2×10^5^) were transfected with control plasmid or plasmids expressing GP73 at different concentrations as indicated (0, 0.125, 0.25 or 0.5 μg), β-actin (0.05 μg), and TRAF3 (0.5 μg) or STING (0.5 μg) for 24 h. Whole cell lysates were subjected to WB with the indicated antibodies. (TIF) [file ppat.1006321.s005.tif]

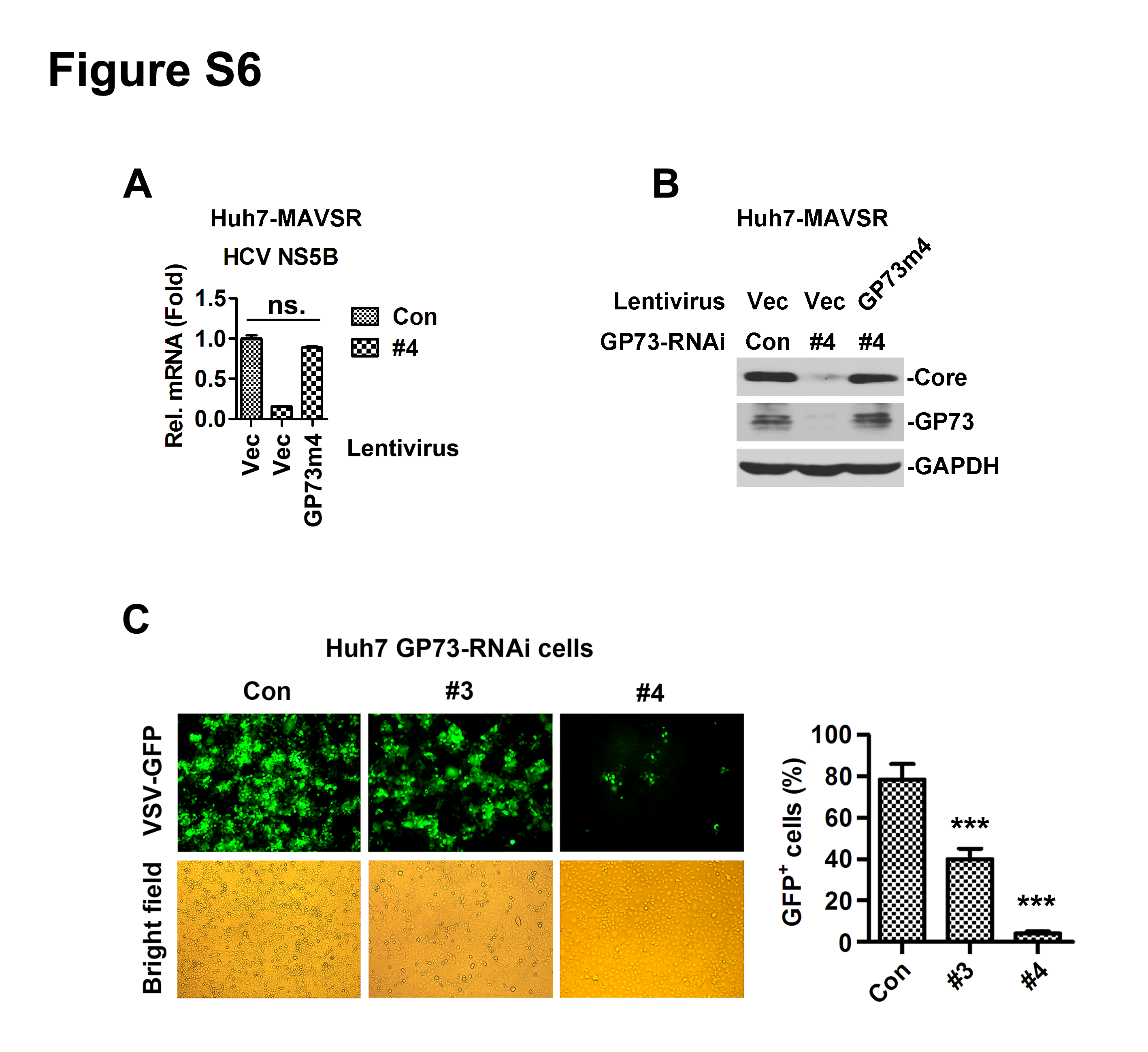

Supplement: S6 Fig — (A, B) Huh7-MAVSR-GP73-RNAi cells were transduced with lentivirus-Vec or lentivirus-GP73m4 for 48 h, followed by HCV infection at MOI = 2 for 3 days. HCV RNAs were determined by RT-PCR (A) and HCV core protein was detected by WB (B). (C) The Huh7-GP73-RNAi cells were plated and infected with VSV-GFP (MOI = 1) for 12 h, followed by analyzing and counting the GFP-positive cells under a fluorescence microscope. ***p < 0.001 compared with control group. (TIF) [file ppat.1006321.s006.tif]
