# Supplementary material for: GP73 represses host innate immune response to promote virus replication by facilitating MAVS and TRAF6 degradation
Source: PLoS Pathog. 2017 Apr 10;13(4):e1006321. doi: 10.1371/journal.ppat.1006321 (PMC5398727; doi:10.1371/journal.ppat.1006321)
Supplement: S1 Table — (DOC) [file ppat.1006321.s007.doc]

**Supplemental Table**

**S1 Table. Primers used in this study.**

| **Gene** | **Forward primer (5’-3’)** | **Reverse primer (5’-3’)** |
| --- | --- | --- |
| GAPDH | GACAAGCTTCCCGTTCTCAG | GAGTCAACGGATTTGGTCGT |
| GP73 | TTGGTAACAGCAAGTCCCAGACA | ACCACCTGGATCTCATTGGTTTC |
| NS5B | TCGTATGATACCCGATGCT | GTTTGACCCTTGCTGTTGA |
| IFNB1 | GATTCATCTAGCACTGGCTGG | CTTCAGGTAATGCAGAATCC |
| IFNL1 | CACATTGGCAGGTTCAAATCTCT | CCAGCGGACTCCTTTTTGG |
| ISG56 | TCACAGGTCAAGGATAGTC | CCACACTGTATTTGGTGTCTAGG |
| MxA | GCCGGCTGTGGATATGCTA | TTTATCGAAACATCTGTGAAAGCAA |
| IL-6 | TTCTCCACAAGCGCCTTCGGTC | TCTGTGTGGGGCGGCTACATCT |
| TNFA | GCCGCATCGCCGTCTCCTAC | CCTCAGCCCCCTCTGGGGTC |
| MAVS | CAGGCCGAGCCTATCATCTG | GGGCTTTGAGCTAGTTGGCA |
| TRAF6 | TTTGCTCTTATGGATTGTCCCC | CATTGATGCAGCACAGTTGTC |
